# Serum Irisin level is associated with fall risk, muscle strength, and cortical porosity in postmenopausal women

**DOI:** 10.3389/fendo.2023.1096950

**Published:** 2023-02-28

**Authors:** Hanting Liang, Wenting Qi, Ruizhi Jiajue, Yue Chi, Wei Liu, Ou Wang, Mei Li, Xiaoping Xing, Wei Yu, Yan Jiang, Weibo Xia

**Affiliations:** ^1^ Department of Endocrinology, Key Laboratory of Endocrinology, National Commission of Health, State Key Laboratory of Complex Severe and Rare Diseases, Peking Union Medical College Hospital, Chinese Academy of Medical Sciences and Peking Union Medical College, Beijing, China; ^2^ Department of Radiology, Peking Union Medical College Hospital, Chinese Academy of Medical Sciences and Peking Union Medical College, Beijing, China

**Keywords:** irisin, postmenopausal women, muscle strength, fall risk, bone microarchitecture

## Abstract

**Background:**

Irisin plays a role in bone-muscle crosstalk, but the relationship between the serum irisin level and bone microarchitecture remains unknown.

**Objective:**

This study aimed to investigate the relationships between serum irisin level and fall risk, muscle strength, bone mineral density (BMD), and bone microarchitecture among Chinese postmenopausal women.

**Methods:**

In all 138 postmenopausal women, handgrip strength, short physical performance battery (SPPB), and the timed up-and-go test were performed to evaluate muscle strength, physical performance, and fall risk, respectively. The serum irisin was measured. High-resolution peripheral quantitative computed tomography (HR-pQCT) was performed to acquire volumetric BMD and bone microarchitecture. Bivariate analysis was used to explore relationships between serum irisin level and muscle strength and HR-pQCT parameters. Univariate and multivariate linear regression analyses were performed to determine associations between serum irisin level and vBMD and cortical porosity (Ct.Po).

**Results:**

All participants had a median serum irisin level of 3.91 μg/ml. Participants with high fall risk had significantly lower serum irisin levels than those with low fall risk (2.22 μg/ml vs. 4.16 μg/ml, p=0.024). Irisin level was positively related to handgrip strength (rs=0.185, p=0.030) and SPPB performance. In univariate linear regression, serum irisin level was positively associated with cortical volumetric BMD (Ct.vBMD, radius: standardized β=0.184, p=0.031; tibia: standardized β=0.242, p=0.004), but it had no significant associations with Ct.vBMD after multivariate adjustment. After adjusting by age, height, serum sclerostin level, and body fat ratio, only Ct.Po at the distal radius had a significantly negative association with serum irisin level (standardized β=-0.276, p=0.003).

**Conclusion:**

Postmenopausal women with lower serum irisin levels have a higher fall risk, weaker muscle strength, and higher cortical porosity. Moreover, serum irisin level has a positive association with Ct.vBMD, but it is affected by factors such as age.

## Introduction

Menopause is a natural event for women during their lifespan caused by the cessation of spontaneous menses or ovariectomy with estrogen decreasing and androgen increasing in circulation (1–3). Body composition changes during the menopause transition, including fat redistribution from extremities to the trunk, bone mineral density (BMD) decrease and bone structure deterioration, and sarcopenia due to muscle mass decrease and muscle function degeneration (4–6). The prevalence of osteoporosis and sarcopenia are 32.1% and approximately 4% in postmenopausal women, respectively (7–9). Osteoporosis and sarcopenia are closely related to the increasing occurrence of falls and fractures, and these two chronic conditions increase fragility and mortality, impact quality of life, and heavy the economic burden among postmenopausal women, especially in those aging postmenopausal women (10, 11). So it is of great significance to identify those individuals with a high risk of falls and fractures by early screening and diagnosis of osteoporosis and sarcopenia among postmenopausal women. Although fasting plasma glucose (FPG) has a positive association with fall occurrence in elderly osteosarcopenic men (12), there is still no specific biomarker to predict the occurrence of osteoporosis/sarcopenia or fractures/falls independently for clinical application.

Irisin is a myokine derived from the fibronectin type III domain-containing protein 5 (FNDC5) protein, which is abundantly expressed in skeletal muscles and can maintain glucose homeostasis and increase energy expenditure by stimulating uncoupling protein-1 expression and converting white fat to brown fat in rodents (13, 14). Irisin plays a role in muscle, adipose, and bone, and it is associated with metabolic syndrome, including diabetes mellitus, obesity, insulin resistance, lipid metabolism disorder, and metabolic bone disorders (15–17). Several studies have explored the relationships between serum irisin level and body composition, falls, and fractures among postmenopausal women. The low circulating irisin level has been proven to be correlated with sarcopenia, osteoporosis, and the history of osteoporotic fractures in postmenopausal women (18–21), but whether it is associated with body composition of lean mass, fat mass, and BMD remains controversial (18, 20).

So far, studies about the relationship between serum irisin level and volumetric bone mineral density (vBMD), bone microarchitecture, falls, and fall risk in postmenopausal women have not been reported yet. This study aimed to explore the relationship between serum irisin level and falls, fractures, muscle strength, and body composition among Chinese community-dwelling postmenopausal women. Meanwhile, we also investigated whether the serum irisin level was associated with parameters evaluated by high-resolution peripheral quantitative computed tomography (HR-pQCT), including bone geometry, vBMD, bone microarchitecture, and estimated bone strength.

## Subjects and methods

### Subjects and study design

This is a cross-sectional study in community-dwelling postmenopausal women based on the Beijing subgroup of the Chinese Vertebral Osteoporosis Study (ChiVos). From September 2021 to December 2021, 138 participants were enrolled. The inclusion criteria included “age more than 50 years old”, “lived in a Beijing urban community for over a half year”, and “no menstruation for at least one year by self-reporting or at least six months after bilateral oophorectomy”. Exclusion criteria: (1) non-Asian; (2) cognitive impairment or physical dysfunction. The study followed the Declaration of Helsinki with the approval of the Ethics Committee of Peking Union Medical College Hospital (JS-2905). All participants were fully informed and signed the consent forms.

### Clinical information collection

General information was collected by the interviewer-administered questionnaire, including date of birth, menopausal age, lifestyle, medication for more than three months (glucocorticoid, hormone replacement therapy, vitamin D and calcium supplementation, and anti-osteoporotic drugs), comorbidities, falls, and fracture history after 50 years old and during the past one year. Falls were based on self-report from participants, and fractures were confirmed by X-rays. Smoking habit is defined as smoking at least one cigarette per day for six months or more. Alcohol intake is defined as one unit of alcohol per week for more than six months. Measuring and recording their height, weight, and waistline, and then calculating body mass index (BMI) and waist-height ratio.

### Physical performance evaluation

Handgrip strength, short physical performance battery (SPPB), and the timed up-and-go (TUG) test were performed to evaluate muscle strength, physical performance, and fall risk, respectively. Handgrip strength was measured by using an electronic handgrip dynamometer (SENSSUM, China). Participants were asked to stand straight and keep their arms in a neutral position, and handgrip strength was measured with each arm three times, finally recording the maximum handgrip strength. SPPB tests were performed as the standard procedure guided (22), consisting of three tests, including the standing balance test, 2.44-meter gait speed test, and repeated rising from a chair test. Each test was scored from 0 to 4, and the total score ranged from 0~12. A higher SPPB score corresponded to better physical performance. The SPPB rank 1 to 4 was corresponding to the SPPB score of 10~12, 7~9, 4~6, and 0~3, respectively. The procedure of the TUG test was as follows: participants were observed and timed since they stand from an armchair, walked 3 meters, turned, walked back, and sat down again (23). The participant who spent more than 12 seconds finishing the TUG test was assessed as having a high risk of falls, otherwise assessed as having a low risk of falls.

### Biochemical measurements

Fasting blood of all participants was collected at 7~8 am, and serum was separated by centrifugation for 10 minutes at 1000×*g*. Briefly, alanine aminotransferase (ALT), FPG, total cholesterol (TC), total triglyceride (TG), low-density lipoprotein cholesterol (LDL-C), high-density lipoprotein cholesterol (HDL-C), creatinine (Cr), calcium, phosphate, and alkaline phosphatase (ALP) were measured by an auto-analyzer (Beckman Coulter AU5800, USA). Parathyroid hormone (PTH) was detected by an autoanalyzer (Beckman Coulter DXI800, USA). Total 25-hydroxyvitamin D (T25OHD), procollagen type 1 N-terminal propeptide (P1NP), C-terminal cross-linking telopeptide of type I collagen (β-CTX), and osteocalcin were detected by the electro-chemiluminescence immunoassay method (Roche Cobas, E601 analyzer, Roche Diagnostics, Switzerland). All biochemical parameters above were detected by using fresh serum, and the rest serum was frozen at -80°C for measurements of irisin and sclerostin. Serum irisin (Cat. No. AG-45A-0046YEK-KI01, AdipoGen, Switzerland, intra-assay coefficient of variation (CV)≤8.2%, inter-assay CV ≤ 9.8%) and sclerostin (Cat. No. BI-20472, BIOMEDICA, Austria, intra-assay CV ≤ 7%, inter-assay CV ≤ 10%) were measured by enzyme-linked immunosorbent assay (ELISA) kits as the manufacturer’s protocol guided.

### Radiological assessment

According to the semi-quantitative method proposed by Genant et al. (24), two experienced radiologists assessed morphometric vertebral fractures (VFs) on the lateral thoracic and lumbar spine X-rays of participants.

All participants accepted the dual-energy X-ray absorptiometry (DXA) evaluation by GE-Lunar scanners (GE-Healthcare, Madison, USA). Parameters of body composition were analyzed by the software enCOREl0.50.086. The body composition parameters included total fat mass (FM), FM percentage, android to gynoid FM ratio (A/G FM), limbs/trunk FM ratio (L/T FM), total lean mass (LM), LM percentage, LM of the upper extremities, leg, and trunk, appendicular lean mass (ALM), and bone mineral density (BMD) of the total body, lumbar vertebrae 1-4 (L1-4), femoral neck (FN), and total hip (TH). Fat mass index (FMI), lean mass index (LMI), and appendicular lean mass index (ALMI) were calculated by FM, LM, and ALM dividing the square of height, respectively. Ratios of leg LM/ALM, ALM/total LM, trunk LM/total LM, and FM/LM were calculated as well. T scores of BMD of the total body, L1-4, FN, and TH were calculated according to the reference ranges of Chinese females. T scores of BMD≤-2.5 at L1-4 or FN or TH, or a T score of “-1~-2.5” at these three sites combined with osteoporotic fractures of the proximal humerus, pelvis, or distal forearm, or previous osteoporotic fractures at the hip or spine were defined as “osteoporosis”. “T score between -2.5 and -1.0” and “T score ≥ -1.0” at the three sites were grouped into “osteopenia” and “normal bone mass”, respectively. According to “2019 Consensus of Asian Working Group for Sarcopenia”, ALMI<5.4 kg/m^2^ combined with low muscle strength (handgrip strength<18 kg= or low physical performance (SPPB score ≤ 9 or TUG time≥12 s) was defined as sarcopenia (25).

According to the previous protocol described, HR-pQCT (XtremeCT II scanner, ScancoMedical, Brüttisellen, Switzerland) with a resolution of 61 μm was performed in the non-dominant distal radius and distal tibia of all participants (26). HR-pQCT parameters included, total area (Tot.Ar), trabecular area (Tb.Ar), cortical area (Ct.Ar), cortical perimeter (Ct.Pm), total vBMD (Tot.vBMD), trabecular vBMD (Tb.vBMD), cortical vBMD (Ct.vBMD), trabecular bone volume fraction (Tb.BV/TV), trabecular number (Tb.N), trabecular thickness (Tb.Th), trabecular separation (Tb. Sp), trabecular inhomogeneity of Network (Tb.1/N.SD), cortical porosity (Ct.Po), cortical thickness (Ct.Th), and cortical pore diameter (Ct.Po.Dm). Estimated bone strength was calculated by Scanco Finite Element software (vision 1.13; Scanco Medical), including stiffness and failure load.

### Statistical analysis

SPSS version 24.0 was used for statistical analysis. The distribution of all continuous variables was determined by the Kolmogorov-Smirnov test. Normally distributed data were depicted as mean ± standard deviation, and non-normally distributed data were shown as median (interquartile, IQR). According to the tertiles of serum irisin level, participants were separated into three groups. Among the three groups, the normally distributed data were compared by the one-way ANOVA test, and the abnormally distributed data were compared by the Kruskal-Wallis H test. The Chi-square test or Fisher’s exact test was used to compare the classified data among the three groups. Serum irisin level was compared by Mann-Whitney U test between the two groups with and without high fall risk/falls/fractures. In bivariate analysis, the continuous variables and the ordered variables were analyzed by Spearman analysis and Kendall’s tau-b analysis, respectively. Univariate and multivariate linear regression analyses were performed to determine the relationships between the serum irisin level and Tot.vBMD, Tb.vBMD, Ct.vBMD, and Ct.Po. P value<0.05 was assigned as a statistically significant difference.

## Results

### General information and biochemical characteristics of the cohort

All general characteristics and biochemical parameters of all participants were listed in **Table 1**. The median record age was 69.0 (14.0) years old, and the median menopausal age was 50.0 (5.0) years old. The medians of height, weight, and BMI were 154.5 (8.5) cm, 60.0 (14.0) kg, 25.57 (5.03) kg/m^2^, respectively. Approximately 3% of participants were sarcopenia, and proportions of osteoporosis and osteopenia accounted for 23.2% and 42.0%, respectively. Over 50% of participants had fall histories after 50 years old, and 23.9% fell in the past year. Those who had fractures after 50 years old and in the recent year accounted for 28.3% and 4.4%, respectively. As for biochemical parameters, the serum irisin of all participants was 3.91 (4.16) μg/ml. The median of T25OHD was 21.4 (10.7) ng/ml, and 44.2% (61/138) of them were diagnosed with vitamin D deficiency.

**Table 1:** General characteristics and biochemical parameters among postmenopausal women according to tertiles of serum irisin levels.

	All (N=138)	T1 (n=46)	T2 (n=46)	T3 (n=46)	p
General characteristics					
**Age (years)**	69.0 (14.0)	77.5 (16.0)	73.0 (14.0)	69.0 (5.0)	**<0.001^a^**
**Height (cm)**	154.5 (8.5)	154.0 (5.9)	154.8 (8.6)	155.6 (9.1)	0.414
**Weight (kg)**	60.0 (14.0)	60.0 (13.3)	59.8 (14.8)	61.5 (16.8)	0.468
**Waistline (cm)**	91.0 (16.5)	88.9 (12.5)	90.0 (15.3)	93.5 (17.8)	0.202
**BMI (kg/m^2^)**	25.57 (5.03)	25.63 (3.85)	25.31 (6.20)	25.85 (5.53)	0.599
**Waist-height ratio**	0.59 ± 0.07	0.58 ± 0.06	0.58 ± 0.07	0.61 ± 0.08	0.173
**Menopausal age (years)**	50.0 (5.0)	50.0 (5.0)	50.0 (4.0)	50.0 (5.0)	0.715
**Smoking**	4.4% (6/136)	4.5% (2/44)	4.3% (2/46)	4.3% (2/46)	>0.999
**Drinking**	3.1% (4/131)	0% (0/44)	2.3% (1/43)	6.8% (3/44)	0.224
**Used/using glucocorticoid (≥ 3 months)**	5.1% (7/136)	8.9% (4/45)	0% (0/45)	6.5% (3/46)	0.129
**HRT (≥ 3 months)**	5.8% (8/138)	6.5% (3/46)	8.7% (4/46)	2.2% (1/46)	0.533
**VitD and calcium supplementation (≥ 3 months)**	30.4% (42/138)	32.6% (15/46)	39.1% (18/46)	19.6% (9/46)	0.130
**Anti-osteoporotic drugs (≥ 3 months)**	15.2% (21/138)	17.4% (8/46)	21.7% (10/46)	6.5% (3/46)	0.140
**Diabetes**	28.7% (39/136)	23.9% (11/46)	21.7% (10/46)	40.9% (18/44)	0.097
**Sarcopenia**	2.9% (4/137)	2.2% (1/45)	2.2% (1/44)	4.3% (2/44)	>0.999
**Percentage of osteoporosis/osteopenia**					0.289
**Osteoporosis**	23.2% (32/138)	30.4% (14/46)	26.1% (12/46)	13.0% (6/46)	
**Osteopenia**	42.0% (58/138)	34.8% (16/46)	39.1% (18/46)	52.2% (24/46)	
Fall and fracture history					
**Falls after 50 years old**	52.2% (72/138)	50.0% (23/46)	54.3% (25/46)	52.2% (23/46)	0.976
**Falls in recent one year**	23.9% (33/138)	17.4% (8/46)	28.3% (13/46)	26.1% (12/46)	0.537
**Fractures after 50 years old**	28.3% (39/138)	37.0% (17/46)	23.9% (11/46)	23.9% (11/46)	0.337
**Fractures in recent one year**	4.4% (6/136)	6.8% (3/44)	4.3% (2/46)	2.2% (1/46)	0.527
**Vertebral fractures**	21.9% (30/137)	21.7% (10/46)	24.4% (11/45)	19.6% (9/46)	0.853
Biochemical parameters					
**ALT (U/L)**	18.0 (11.0)	18.0 (11.0)	16.0 (9.0)	20.0 (12.0)	**0.035^b^**
**FPG (mmol/L)**	5.7 (1.7)	5.7 (1.3)	5.7 (1.3)	5.8 (3.2)	0.766
**TC (mmol/L)**	4.79 (1.27)	4.81 (1.41)	4.78 (1.04)	4.96 (1.43)	0.540
**TG (mmol/L)**	1.39 (0.87)	1.31 (0.75)	1.27 (0.78)	1.54 (0.93)	0.118
**LDL-C (mmol/L)**	2.67 (1.16)	2.67 (1.20)	2.67 (1.12)	2.70 (1.34)	0.555
**HDL-C (mmol/L)**	1.31 (0.44)	1.29 (0.45)	1.37 (0.60)	1.30 (0.30)	0.677
**Cr (μmol/L)**	64.0 (18.0)	64.5 (22.0)	64.0 (15.0)	62.5 (15.0)	0.133
**Ca (mmol/L)**	2.35 (0.11)	2.37 (0.13)	2.34 (0.09)	2.32 (0.12)	0.076
**P (mmol/L)**	1.19 (0.22)	1.20 (0.25)	1.20 (0.22)	1.18 (0.22)	0.810
**ALP (U/L)**	79.5 (22.0)	79.5 (22.0)	77.0 (20.0)	83.0 (26.0)	0.302
**T25OHD (ng/ml)**	21.4 (10.7)	21.1 (13.4)	21.5 (11.1)	21.1 (7.9)	0.802
**β-CTX (ng/ml)**	0.40 (0.23)	0.41 (0.32)	0.41 (0.24)	0.40 (0.22)	0.547
**P1NP (ng/ml)**	46.8 (25.4)	43.5 (24.8)	50.3 (25.1)	46.4 (25.1)	0.289
**PTH (pg/ml)**	47.35 (26.70)	47.60 (32.12)	46.45 (24.93)	48.05 (25.80)	0.863
**Osteocalcin (ng/ml)**	17.0 (7.0)	16.0 (7.0)	18.5 (6.0)	16.0 (8.0)	0.136
**Sclerostin (pmol/L)**	80.14 (39.77)	78.88 (44.07)	75.52 (41.12)	85.32 (32.76)	0.866

All subjects were grouped according to tertiles of serum irisin levels. T1: 1.171~2.802 μg/ml; T2: 2.802~5.796 μg/ml; T3: 5.796~11.632 μg/ml. All continuous variables were described as median (interquartile). a: p<0.05 between the T1 and T3 groups; b: p<0.05 between the T2 and T3 groups. BMI, body mass index; HRT, hormone replacement therapy; VitD, vitamin D; ALT, alanine aminotransferase; FPG, fasting plasma glucose; TC, total cholesterol; TG, total triglyceride; LDL-C, low-density lipoprotein cholesterol; HDL-C, high-density lipoprotein cholesterol; Cr, creatinine; Ca, calcium; P, phosphorus; ALP, alkaline phosphatase; T25OHD, total 25-hydroxyvitamin D; β-CTX, C-terminal cross-linking telopeptide of type I collagen; P1NP, procollagen type 1 N-terminal propeptide; PTH, parathyroid hormone.

According to the tertiles of the serum irisin level, we divided all participants into three groups, which were the first tertile (T1: 1.171~2.802 μg/ml), the second tertile (T2: 2.802~5.796 μg/ml), and the third tertile (T3: 5.796~11.632 μg/ml) groups. Compared to the T3 group, participants’ age in the T1 group was significantly higher (p<0.05). Other general characteristics, proportions of sarcopenia/osteoporosis/osteopenia, histories of falls, clinical fractures, and VFs had no significant differences among the three groups. Except for ALT, other biochemical parameters had no significant differences among the three groups.

### Serum irisin level in postmenopausal women with and without high fall risk/falls/fractures

**Figure 1:**
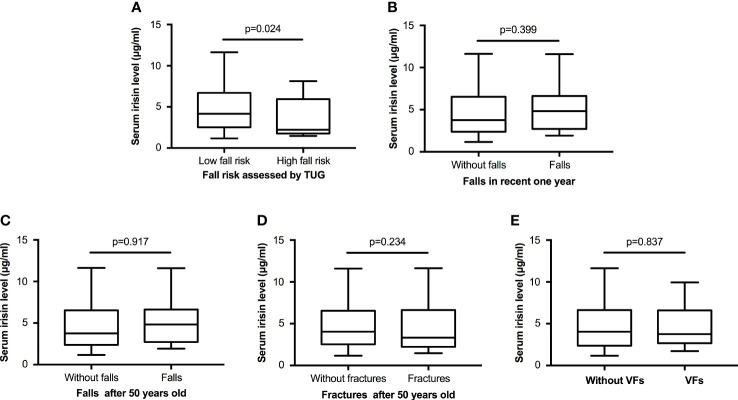
The serum irisin level in postmenopausal women with and without high fall risk/falls/fractures. **(A)** Comparison of the serum irisin level between the low and high fall risk groups according to the TUG test. **(B, C)** Comparison of the serum irisin level between the two groups without and with falls in recent one year and after 50 years old. **(D)** Comparison of the serum irisin level between the two groups without and with fractures after 50 years old. **(E)** Comparison of the serum irisin level between the two groups without and with previous VFs. All comparison above was performed by the Mann-Whitney U test. TUG, timed up and go test; VFs, vertebral fractures.

According to whether the finish time of TUG was more than 12 seconds, we separated participants into two groups, which were “low fall risk” (n=117) and “high fall risk” (n=16). Participants with high fall risk had significantly lower serum irisin levels than those with low fall risk, which were 2.22 (4.17) μg/ml vs. 4.16 (4.19) μg/ml, p=0.024 (**Figure 1A**). However, the serum irisin level had no statistically significant differences between groups with and without falls, with and without fractures, and with and without VFs (**Figures 1B–E**; **Supplementary Table 1**).

### Correlations between serum irisin level and body composition and muscle strength

**Figure 2:**
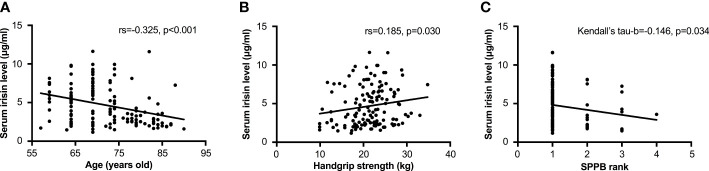
Relationships between the serum irisin level and age and muscle function. **(A)** Correlation between the serum irisin level and age by Spearman analysis. **(B)** Correlation between the serum irisin level and handgrip strength by Spearman analysis. **(C)** Correlation between the serum irisin level and age by Kendall’s tau-b analysis. The SPPB rank 1 to 4 was corresponding to the SPPB score of 10~12, 7~9, 4~6, and 3~0, respectively. SPPB, short-physical performance battery; TUG, timed up and go test.

In bivariate analysis, age was negatively associated with the serum irisin level (rs=-0.325, p<0.001, **Figure 2A**). There was no correlation between serum irisin level with BMI (rs=0.032, p=0.708). The associations between serum irisin level and body composition were shown in **Table 2**. Except that L/T FM had a significantly negative association with serum irisin level (rs=-0.201, p=0.018), other body composition had no significant relationships with serum irisin level, including body fat, muscle-related parameters, and BMD. Serum irisin level was also significantly associated with muscle strength and physical performance. It had a positive correlation with handgrip strength (rs=0.185, p=0.030, **Figure 2B**) and a negative correlation with SPPB rank (Kendall’s tau-b=-0.146, p=0.034, **Figure 2C**).

**Table 2:** Bivariate correlation analysis between serum irisin level and body composition.

	rs	p
**FM percentage (%)**	0.033	0.702
**Total FM (kg)**	0.046	0.596
**FMI (kg/m^2^)**	0.036	0.672
**A/G FM**	0.081	0.342
**L/T FM**	**-0.201**	**0.018**
**LM percentage (%)**	-0.036	0.676
**Total LM (kg)**	0.053	0.536
**LMI (kg/m^2^)**	0.034	0.693
**Upper extremities LM (kg)**	0.050	0.562
**Leg LM (kg)**	0.044	0.612
**Trunk LM (kg)**	0.035	0.685
**ALM (kg)**	0.048	0.574
**ALMI (kg/m^2^)**	0.031	0.715
**Leg LM/ALM**	-0.018	0.831
**ALM/total LM**	-0.021	0.811
**Trunk LM/total LM**	-0.018	0.837
**FM/LM**	0.032	0.709
**Total body BMD (g/cm^2^)**	0.124	0.146
**T score of total body BMD**	0.121	0.156
**BMD at FN (g/cm^2^)**	0.078	0.361
**T score of BMD at FN**	0.136	0.111
**BMD at TH (g/cm^2^)**	0.136	0.111
**T score of BMD at TH**	0.126	0.141
**BMD at L1-4 (g/cm^2^)**	0.110	0.205
**T score of BMD at L1-4**	0.101	0.244

Continuous variables were analyzed by Spearman analysis. FM, fat mass; FMI, fat mass index; A/G FM, android to gynoid FM ratio; L/T FM, limbs/trunk FM ratio; LM, lean mass; LMI, lean mass index; ALM, appendicular lean mass; ALMI, appendicular lean mass index; BMD, bone mineral density; FN, femoral neck; TH, total hip; L1-4, lumbar vertebrae 1-4.

### Correlations between serum irisin level and HR-pQCT parameters

Bivariate correlations between serum irisin level and parameters evaluated by HR-pQCT were displayed in **Table 3**. Parameters of bone geometry at the distal radius and tibia did not correlate with serum irisin level. As for vBMD, serum irisin level was positively correlated with Tot.vBMD (rs=0.173, p=0.043) and Ct.vBMD (rs=0.215, p=0.011) at the distal radius, but only had a positive correlation with Ct.vBMD (rs=0.213, p=0.012) at the distal tibia. Trabecular bone microarchitecture had no significant correlations with serum irisin level at both sites, including Tb.BV/TV, Tb.N, Tb.Th, Tb.Sp, and Tb.1/N.SD. However, both at the distal radius and tibia, Ct.Po was negatively associated with serum irisin level (radius: rs=-0.219, p=0.010; tibia: rs=-0.223, p=0.009). Ct.Th, Ct.Po.Dm, and estimated bone strength at both sites did not correlate with serum irisin level.

**Table 3:** Correlation analysis between the serum irisin level and parameters evaluated by HR-pQCT.

	Radius		Tibia	
	rs	p value	rs	p value
Bone geometry				
**Tot.Ar (mm^2^)**	-0.125	0.142	-0.098	0.255
**Tb.Ar (mm^2^)**	-0.154	0.070	-0.112	0.193
**Ct.Ar (mm^2^)**	0.155	0.069	0.092	0.284
**Ct.Pm (mm)**	-0.139	0.105	-0.109	0.204
Volumetric bone mineral density				
**Tot.vBMD (mg HA/cm^3^)**	**0.173**	**0.043**	0.164	0.056
**Tb.vBMD (mg HA/cm^3^)**	0.078	0.363	0.089	0.300
**Ct.vBMD (mg HA/cm^3^)**	**0.215**	**0.011**	**0.213**	**0.012**
Bone microarchitecture				
**Tb.BV/TV**	0.056	0.513	0.078	0.365
**Tb.N (1/mm)**	0.088	0.303	0.108	0.209
**Tb.Th (mm)**	-0.085	0.321	-0.017	0.846
**Tb.Sp (mm)**	-0.093	0.280	-0.119	0.166
**Tb.1/N.SD (mm)**	-0.097	0.260	-0.115	0.182
**Ct.Po**	**-0.219**	**0.010**	**-0.223**	**0.009**
**Ct.Th (mm)**	0.151	0.076	0.097	0.258
**Ct.Po.Dm (mm)**	-0.058	0.501	0.045	0.604
Estimated bone strength				
**Stiffness (kN/mm)**	0.083	0.334	0.089	0.302
**Failure load (kN)**	-0.097	0.259	-0.083	0.336

HR-pQCT, high-resolution peripheral quantitative computed tomography; Tot.Ar, total area; Tb.Ar, trabecular area; Ct.Ar, cortical area; Ct.Pm, cortical perimeter; vBMD, volumetric bone mineral density; Tot.vBMD, total vBMD; Tb.vBMD, trabecular vBMD; Ct.vBMD, cortical vBMD; Tb.Th, trabecular thickness; Tb. Sp, trabecular separation; Tb.N, trabecular number; Tb.BV/TV, trabecular bone volume fraction; Tb.1/N.SD, trabecular inhomogeneity of Network; Ct.Po, cortical porosity; Ct.Th, cortical thickness; Ct.Po.Dm, cortical pore diameter. Bold values denote statistically significant differences.

We performed univariate and multiple linear regression analyses between serum irisin level and vBMD and Ct.Po further (**Table 4**). In univariate linear regression analysis, both at the distal radius and tibia, serum irisin level was positively associated with Ct.vBMD (radius: standardized β=0.184, adjusted r^2^ = 0.027, p=0.031; tibia: standardized β=0.242, adjusted r^2^ = 0.052, p=0.004) and negatively associated with Ct.Po (radius: standardized β=-0.337, adjusted r^2^ = 0.107, p<0.001; tibia: standardized β=-0.228, adjusted r^2^ = 0.045, p=0.008), but did not associate with Tot.vBMD and Tb.vBMD. After adjusting by age, height, serum sclerostin level, and body fat ratio, only Ct.Po at the distal radius had a significantly negative association with serum irisin level (standardized β=-0.276, adjusted r^2^ = 0.119, p=0.003).

**Table 4:** Linear regression analysis between the serum irisin level and vBMDs and Ct.Po.

	Unadjusted			Model 1			Model 2		
	β	Adj-R^2^	p	β1	Adj-R^2^	p1	β2	Adj-R^2^	p2
Radius									
**Tot.vBMD (mg HA/cm^3^)**	0.167	0.021	0.052	-0.068	0.331	0.360	-0.051	0.387	0.479
**Tb.vBMD (mg HA/cm^3^)**	0.084	<0.001	0.328	-0.070	0.199	0.390	-0.083	0.322	0.275
**Ct.vBMD (mg HA/cm^3^)**	**0.184**	**0.027**	**0.031**	-0.009	0.264	0.906	-0.017	0.317	0.824
**Ct.Po**	**-0.337**	**0.107**	**<0.001**	**-0.267**	**0.116**	**0.004**	**-0.276**	**0.119**	**0.003**
Tibia									
**Tot.vBMD (mg HA/cm^3^)**	0.163	0.019	0.057	0.009	0.208	0.915	-0.020	0.346	0.789
**Tb.vBMD (mg HA/cm^3^)**	0.069	<0.001	0.428	-0.039	0.134	0.643	-0.039	0.310	0.616
**Ct.vBMD (mg HA/cm^3^)**	**0.242**	**0.052**	**0.004**	0.109	0.190	0.186	0.083	0.291	0.289
**Ct.Po**	**-0.228**	**0.045**	**0.008**	-0.138	0.082	0.120	-0.111	0.085	0.215

Model 1: adjusted by age. Model 2: adjusted by age, height, serum sclerostin level, and body fat ratio. “β, β1, and β2” are standardized β value. Adj-R^2^, adjusted r-square; vBMD, volumetric bone mineral density; Tot.vBMD, total vBMD; Tb.vBMD, trabecular vBMD; Ct.vBMD, cortical vBMD; Ct.Po, cortical porosity. Bold values denote statistically significant differences.

## Discussion

In this study, we showed that the serum irisin level was correlated with fall risk and muscle strength, but not previous falls or fractures, and most parameters of body composition like fat mass, lean mass, and BMD among postmenopausal women. Meanwhile, we investigated the relationship between the serum irisin level and bone microarchitecture in postmenopausal women for the first time and revealed that serum irisin level was the independent factor of Ct.Po at the distal radius.

Our result showed that postmenopausal women with higher serum irisin levels were younger, and it was consistent with the bivariate analysis of a negative correlation between age and serum irisin level in this study and other studies performed on postmenopausal women (18, 21). So serum irisin level decreases with aging in postmenopausal women. Other general characteristics had no significant differences among the three groups with different serum irisin levels, including proportions of osteoporosis/osteopenia and sarcopenia, and that was not the same as in previous studies. Cross-sectional studies and a meta-analysis showed that postmenopausal women with osteoporosis had significantly lower serum irisin level than those without osteoporosis, indicating serum irisin level was associated with osteoporosis (19, 27, 28). Moreover, low serum irisin level was the independent risk factor of sarcopenia in postmenopausal women (18). However, only 4 participants were sarcopenia in this study, accounting for approximately 3%, which restricted us to explore the association between sarcopenia and irisin level further. Different study populations and study designs might help explain the inconsistent results of these studies.

A statistical difference in ALT between T2 and T3 groups might have no clinical significance. Bone metabolic markers had no differences among the three groups, including calcium, phosphate, ALP, PTH, T25OHD, P1NP, and β-CTX. However, Badr Roomi et al. showed that most of these bone metabolic markers had significant correlations with the irisin level in a group of postmenopausal women (27). Compared with our study, the participants in Badr Roomi et al.’s study were younger and had low T scores of BMD and severe vitamin D deficiency. Correlations between serum irisin level and other myokines and bone-derived cytokines were different. For example, irisin was positively correlated with the osteocalcin level in healthy children (29) but negatively associated with the sclerostin level in adults with pre-diabetes (30). But in this study, these cytokines mentioned above had no differences among the three groups with different irisin levels, as well as no correlation with serum irisin level (data not shown), which might be related to age and health status.

We found that the serum irisin level had no difference in postmenopausal women with and without histories of falls and fractures (including VFs), no matter recent or distant events, which was quite different from previous studies. Former research showed that low serum irisin level was independently associated with fracture risks in postmenopausal women, including hip fractures, vertebral fragility fractures, and previous osteoporotic fractures (20, 21, 31). The difference between our study and these studies might be explained by different study designs, that was, previous studies were case-control studies, but this study was a *post hoc* analysis of a community epidemiological survey, so the study populations were not comparable. To our best knowledge, the association between irisin level and falls had not been reported in postmenopausal women. Our result displayed that serum irisin level was not associated with falls in the recent year or after 50 years old, but the irisin level was low in those with high fall risk evaluated by TUG. Since the half-life period of circulating irisin is unknown and that of the recombinant irisin is less than one hour (32), this phenomenon indicated that irisin might be more suitable for predicting events shortly, such as fall risk.

Both our results and the study performed by Palermo et al. showed no significant correlations between serum irisin level and body composition assessed by DXA in postmenopausal women, including fat mass, lean mass, or BMD (20). However, parameters of muscle mass evaluated by quantitative computed tomography were positively related to serum irisin level in another group of postmenopausal women (18). Intriguingly, the ratio of L/T FM had a slightly negative correlation with serum irisin level, indicating that the distribution of fat tissue might affect the production and secretion of irisin.

However, serum irisin level had a relatively satisfactory relationship with muscle strength. Consistent with the previous study, serum irisin level was positively related to muscle strength and physical performance in postmenopausal women (18). Although muscle mass was a vital determinant of muscle strength, the decline of muscle strength was more rapid than that of muscle mass in the older population, indicating that muscle quality decreased (33). It might be helpful to explain that serum irisin level was correlated with muscle strength but not muscle mass in this cohort, so we inferred that serum irisin level was more closely related to muscle quality.

As for BMD, serum irisin level was only positively associated with Ct.vBMD at the distal radius and tibia but not aBMD, Tot.vBMD, or Tb.vBMD. After multivariate adjustment, these significantly positive associations no longer exist. Parameters of bone microarchitecture were not correlated with serum irisin level in postmenopausal women, except Ct.Po at the distal radius and tibia was positively associated with serum irisin level, and the positive association still existed at the distal radius after multivariate adjustment. Moreover, serum irisin level was more closely related to cortical bone microarchitecture than trabecular bone microarchitecture, and since Ct.Po was a characteristic marker for predicting additional fracture risk not captured by the Fracture Risk Assessment Tool (FRAX) (34), so it might be inferred that the microarchitecture of cortical bone had been changed at an early stage in postmenopausal women with low serum irisin level due to irisin synthesized by muscles preferentially acted on cortical bone through paracrine.

There are still limitations existing in this study. First, it was a cross-sectional study based on the Beijing subgroup of ChiVos with a limited sample size, which could not explain the causal relationship between the serum irisin level and fall risk and muscle strength, and the conclusion could not be generalized to all postmenopausal women as well. Second, we did not enroll younger postmenopausal women, and the youngest participant in this study was 57 years old, so lacking data from younger postmenopausal women might influence the result. Third, the irisin measurement used the frozen but not fresh serum sample, which might bring bias to the final results due to the short half-life period of circulating irisin, and the method for quantitative assays of serum irisin is still a big challenge (35). Fourth, we did not exclude the influence of daily activity and physical exercise on the serum irisin level, since high-intensity concurrent interval exercise could improve the serum irisin level in postmenopausal women (36–38). In addition, we only evaluated the fall risk by the TUG test but not estimated the fracture risk by the FRAX in all participants.

In conclusion, serum irisin level was low in postmenopausal women with high fall risk and weak muscle strength. The serum irisin level has a positive association with Ct.vBMD, but it is affected by factors such as age. Ct.Po was higher in those with the lower serum irisin level, which might indicate high fracture risk to some extent, but the irisin level was not associated with the microarchitecture of trabecular bone. Moreover, serum irisin level was not correlated with previous falls and fractures, fat mass, muscle mass, and BMD. So the predictive value of serum irisin level on the occurrence of falls and fractures and the prevalence of osteoporosis and sarcopenia needs further exploration in postmenopausal women.

## Data availability statement

The original contributions presented in the study are included in the article/**Supplementary Material**. Further inquiries can be directed to the corresponding authors.

## Ethics statement

The studies involving human participants were reviewed and approved by the Ethics Committee of Peking Union Medical College Hospital. The patients/participants provided their written informed consent to participate in this study.

## Author contributions

WX designed the study. WX and YJ revised the manuscript. HL analyzed the data and draft the manuscript. HL and WQ measured serum levels of irisin and sclerostin in all participants. WY collected X rays and DXA data. RJ and YC collected HR-pQCT data. WL, YJ, OW, ML, XX and WX recruited participants and collected questionnaire of participants. HL, YJ and WX are responsible for the integrity of the data analysis. All authors contributed to the article and approved the submitted version.
