# Supplementary material for: Serum Irisin level is associated with fall risk, muscle strength, and cortical porosity in postmenopausal women
Source: Front Endocrinol (Lausanne). 2023 Feb 28;14:1096950. doi: 10.3389/fendo.2023.1096950 (PMC10013910; doi:10.3389/fendo.2023.1096950)
Supplement: Supplementary file 1 [file Table_1.docx]

**Table 1. Serum irisin level between groups with and without falls/fractures/VFs.**

| **Event** | **Without (μg/ml)** | **With (μg/ml)** | ***p* value** |
| --- | --- | --- | --- |
| **Falls in the past year** | 3.75 (4.14) | 4.83 (3.91) | 0.399 |
| **Falls after 50 years old** | 3.82 (4.22) | 4.05 (4.19) | 0.917 |
| **Fractures after 50 years old** | 4.04 (4.02) | 3.33 (4.39) | 0.234 |
| **VFs** | 4.04 (4.26) | 3.75 (3.92) | 0.837 |

The serum irisin level is depicted as median (interquartile). Abbreviations: VFs, vertebral fractures.
